# Effect of Intravenous Clonidine Premedication on the Attenuation of Hemodynamic Stress Response in Patients Undergoing Laparoscopic Hysterectomy

**DOI:** 10.7759/cureus.112396

**Published:** 2026-07-10

**Authors:** Elambarithy Thangasamy, Sarva Vinothini J

**Affiliations:** 1 Anaesthesiology, MIOT International Hospitals, Chennai, IND

**Keywords:** clonidine, hemodynamic stress response, laparoscopic hysterectomy, pneumoperitoneum, tracheal intubation

## Abstract

Background

Creation of pneumoperitoneum during laparoscopic surgery produces significant hemodynamic changes due to increased intra-abdominal pressure and sympathetic stimulation. Various pharmacological agents have been used to attenuate this stress response. Clonidine, an α2-adrenergic agonist, has sympatholytic properties that may provide hemodynamic stability during surgery.

Aim

This study aimed to evaluate the efficacy of intravenous clonidine in attenuating the hemodynamic stress response during laparoscopic hysterectomy.

Methods

This prospective randomized double-blind placebo-controlled trial was conducted on 90 female patients aged 30-60 years with American Society of Anesthesiologists (ASA) physical status I-II undergoing elective laparoscopic hysterectomy. Patients were randomly allocated into two groups of 45 each. Group I received intravenous clonidine 2 mcg/kg diluted in 100 ml normal saline 15 minutes before induction, while Group II received 100 ml normal saline as a placebo. Hemodynamic parameters, including heart rate (HR), systolic blood pressure (SBP), diastolic blood pressure (DBP), and mean arterial pressure (MAP), were recorded at baseline, after drug administration, post-intubation, during pneumoperitoneum, and after CO₂ release.

Results

Patients receiving clonidine demonstrated significantly lower HR, SBP, DBP, and MAP during pneumoperitoneum compared with the placebo group (p < 0.05). In the placebo group, 12 patients developed hypertension, and eight patients developed tachycardia requiring nitroglycerine infusion and fentanyl administration. In the clonidine group, only three patients developed bradycardia, which responded to atropine. No significant postoperative sedation or nausea and vomiting were observed.

Conclusion

Intravenous clonidine 2 mcg/kg administered 15 minutes before induction effectively attenuates the hemodynamic stress response to pneumoperitoneum during laparoscopic hysterectomy without significant adverse effects.

## Introduction

Laryngoscopy and endotracheal intubation are known to provoke a reflex sympathetic response, resulting in transient increases in heart rate and arterial blood pressure [1,2]. In laparoscopic procedures, these physiological changes are further accentuated by pneumoperitoneum, which increases intra-abdominal pressure and activates neurohumoral pathways [3,4]. The combined effect leads to enhanced catecholamine release and sympathetic stimulation, potentially causing perioperative cardiovascular instability, particularly in patients with pre-existing cardiac conditions [5].

Various pharmacological agents, including opioids, beta blockers, vasodilators, and α2-adrenergic agonists, have been used to attenuate this response [6]. Clonidine, a centrally acting α2-adrenergic agonist, reduces sympathetic outflow and provides sedative as well as analgesic effects [7]. Previous studies have demonstrated that clonidine improves perioperative hemodynamic stability and reduces anesthetic and analgesic requirements in laparoscopic surgeries [8-10].

Laparoscopic hysterectomy is a commonly performed surgical procedure; however, the combined effects of airway manipulation and pneumoperitoneum can result in significant hemodynamic fluctuations [11,12]. Therefore, the present study was undertaken to evaluate the effect of intravenous clonidine premedication on perioperative hemodynamic responses in patients undergoing laparoscopic hysterectomy.

## Materials and methods

Study design and participants

This prospective, randomized, double-blind, placebo-controlled trial was conducted at MIOT International Hospitals between February 2021 and August 2021, with approval from the Scientific Research Committee. Written informed consent was obtained from all participants before enrollment.

Sample size calculation

The sample size was calculated with the assistance of a statistician using G*Power version 3.1.9.7 (Heinrich Heine University, Düsseldorf, Germany). Assuming a two-sided significance level (α) of 0.01, a study power of 95%, and an allocation ratio of 1:1, the minimum required sample size was calculated to be 38 participants in each group. To compensate for potential dropouts and incomplete data, the sample size was increased to 45 participants per group, resulting in a total sample size of 90 patients. 

A total of 90 female patients aged 30-60 years, belonging to American Society of Anesthesiologists (ASA) physical status I and II, and scheduled for elective laparoscopic hysterectomy under general anesthesia were included. Patients with ASA physical status III or IV, poorly controlled hypertension, significant cardiovascular disease, arrhythmias, chronic respiratory illness, those on sedative or antidepressant therapy, or those unwilling to participate were excluded.

Randomization and intervention

Participants were randomly assigned into two equal groups (n = 45 each) using a computer-generated randomization method. Allocation concealment was ensured using sequentially numbered, opaque, sealed envelopes that were opened only after participant enrollment. The study drug was prepared by an anesthesiologist who was not involved in patient management or outcome assessment, thereby maintaining blinding of both the treating anesthesiologist and the outcome assessor. Hemodynamic variables were measured using standardized monitoring equipment at predefined time points. All prespecified outcomes were analyzed and reported, thereby minimizing the risk of selective reporting bias.

Group I (Clonidine group): Received intravenous clonidine 2 µg/kg diluted in 100 mL normal saline, administered 15 minutes before induction of anesthesia.

Group II (Control group): Received 100 mL of normal saline intravenously 15 minutes before induction.

The study drug was administered 15 minutes before induction because intravenous clonidine reaches its peak sympatholytic and hemodynamic effect approximately 15-20 minutes after administration, thereby providing optimal attenuation of the pressor response to laryngoscopy, tracheal intubation, and pneumoperitoneum.

Anesthetic technique

Standard monitoring, including electrocardiography, noninvasive blood pressure monitoring, and pulse oximetry, was instituted. Baseline hemodynamic parameters were recorded before induction.

General anesthesia was induced using midazolam 1 mg, fentanyl 2 µg/kg, propofol 2 mg/kg, and vecuronium 0.1 mg/kg to facilitate endotracheal intubation. Anesthesia was maintained with an oxygen-air mixture and isoflurane, along with intermittent doses of vecuronium (0.02 mg/kg) for muscle relaxation.

Controlled mechanical ventilation was adjusted to maintain end-tidal carbon dioxide (EtCO₂) between 35 and 40 mmHg. Pneumoperitoneum was established using carbon dioxide, with intra-abdominal pressure between 12 and 15 mmHg throughout the procedure.

Data collection

Hemodynamic variables, including heart rate (HR), systolic blood pressure (SBP), diastolic blood pressure (DBP), and mean arterial pressure (MAP), were recorded at baseline, after administration of the study drug, immediately after intubation, before pneumoperitoneum, and at predefined intervals during pneumoperitoneum until completion of surgery.

Statistical analysis

Data were analyzed using appropriate statistical software. Normality of continuous variables was assessed using the Shapiro-Wilk test by the study statistician. As the continuous variables were normally distributed (p > 0.05), they were expressed as mean ± standard deviation (SD), and intergroup comparisons were performed using the independent samples Student's t-test. Categorical variables were expressed as frequencies and percentages and compared using the Pearson chi-square test or Fisher's exact test, as appropriate. All statistical tests were two-tailed, and a p-value < 0.05 was considered statistically significant.

## Results

Distribution of patients (n = 90) according to age, weight, and duration of surgery is presented in Table 1. There were no statistically significant differences between the groups with respect to these baseline characteristics. The p-values for age (0.858), weight (0.897), and duration of surgery (0.813) were all greater than 0.05, indicating that the groups were comparable. An independent samples t-test was used for the analysis.

**Table 1:** Distribution of groups by age, weight, and duration of surgery Values are expressed as mean ± standard deviation (SD). Group I received intravenous clonidine (2 µg/kg), and Group II received normal saline (placebo). An independent samples t-test was used for comparison between the two groups. A p-value < 0.05 was considered statistically significant.

Variables	Group - I				Group - II				Test Statistic	P-value
	Mean	± SD	Min	Max	Mean	± SD	Min	Max	t	
Age	49.2	5.8	37.0	59.0	49.5	5.9	37.0	59.0	-0.24	0.858
Weight	63.5	8.2	45.0	85.0	63.7	8.2	45.0	85.0	-0.11	0.897
Duration of surgery	121.8	8.1	110.0	140.0	121.4	8.4	105.0	140.0	0.23	0.813

The distribution of patients according to ASA grades is presented in Table 2. There was no statistically significant difference between the groups (Group I, n = 45; Group II, n = 45) with respect to ASA grade distribution (p = 0.378), indicating that the groups were comparable. The chi-square test was used for analysis.

**Table 2:** Distribution of groups by American Society of Anesthesiologists (ASA) grade Data are expressed as frequencies and percentages. Pearson's Chi-square test showed no statistically significant difference between the groups (p > 0.05).

	ASA Grade				
Group	I	II	N	Test statistic (X^2^)	P-value
Group - I	27	18	45	0.778	0.378
%	60.0%	40.0%	100.0%		
Group - II	31	14	45		
%	68.9%	31.1%	100.0%		
N	62	28	90		

Measurements were recorded at baseline; 15 minutes after administration of the study drug (IV clonidine or IV normal saline); post-intubation; before CO₂ insufflation; and at 5, 15, and 30 minutes after CO₂ insufflation, followed by every 30 minutes until release of CO₂ pneumoperitoneum (PNO). The independent samples t-test was used to calculate p-values for comparisons between the groups.

Comparison of HR between Group I (clonidine) and Group II (normal saline) at various time intervals is shown in Table 3. Baseline HR was comparable between the two groups (p > 0.05). Following drug administration, HR remained significantly lower in Group I compared to Group II at multiple time intervals. During laryngoscopy and tracheal intubation, Group I showed a significant reduction in HR compared to baseline (p = 0.013), whereas Group II demonstrated an increase in HR above baseline values. Similarly, during PNO (CO₂ insufflation), HR was significantly lower in Group I (p < 0.001), while Group II showed a sustained increase throughout the study period. At all measured intervals, HR values were significantly lower in Group I compared to Group II (p < 0.05). Bradycardia was observed in three patients in Group I and was managed with intravenous atropine 0.6 mg. Tachycardia occurred in eight patients in Group II and was treated with intravenous fentanyl 50 µg.

**Table 3:** Heart rate recorded in both groups at specific time points Data are presented as mean ± SD. Independent samples t-test showed significantly lower heart rate in Group I compared to Group II at multiple intraoperative time points (p < 0.05), while baseline values were comparable.

Heart rate	Group - I		Group - II		Test statistic (t)	P-value
	Mean	± SD	Mean	± SD		
Baseline	77.36	6.59	77.73	7.42	-0.25	0.799
15 min of IV drug	71.64	6.44	77.51	7.42	-4.01	0.021
Post-intubation	75.76	6.59	80.02	6.60	-3.06	0.013
Prior to CO2 insufflation	74.73	6.36	85.80	5.91	-8.55	0.024
5 min after CO2 insufflation	76.27	6.59	88.76	6.25	-10.69	0.017
15 min after CO2 insufflation	76.69	6.65	89.24	5.72	-13.40	0.015
30 min after CO2 insufflation	76.33	7.06	90.73	4.53	-13.15	0.008
60 min after CO2 insufflation	75.80	7.30	91.40	5.29	-11.60	0.005
90 min after CO2 insufflation	74.84	7.14	89.91	5.12	-11.51	<0.001
After release of CO2	70.78	5.19	80.40	7.85	-6.87	<0.001

Comparison of SBP between Group I (clonidine) and Group II (normal saline) at various time intervals is shown in Table 4. Baseline SBP comparison between Group I and Group II was not significant (p > 0.05). In Group I, SBP was well maintained below the baseline value during tracheal intubation (p < 0.02). During PNO insufflation, an increase in SBP was noted; the values approached baseline within 15 to 30 minutes of PNO insufflation and continued to remain within baseline values throughout the duration of PNO (p < 0.05). In Group II, SBP was increased above the baseline value immediately after tracheal intubation and remained increased throughout the study period. The p-value was <0.05 in most of the readings, which was statistically significant. Twelve patients in Group II were treated with nitroglycerin (NTG) infusion for hypertension.

**Table 4:** Systolic blood pressure (SBP) recorded in both groups at specified timings Data are presented as mean ± SD. Independent samples t-test showed significantly lower SBP in Group I compared to Group II at multiple intraoperative time points (p < 0.05), while baseline values were comparable.

Systolic Blood Pressure	Group - I		Group - II		Test Statistic (t)	P-value
	Mean	± SD	Mean	± SD		
Baseline	117.16	7.53	119.53	6.37	-0.85	0.395
15 min of IV drug	112.87	6.38	119.27	6.32	-4.78	0.018
Post-intubation	114.58	6.49	120.49	5.88	-9.13	0.020
Prior to CO2 insufflation	111.71	6.53	122.53	6.34	-6.80	0.017
5 min after CO2 insufflation	118.27	6.45	125.51	4.40	-13.11	0.013
15 min after CO2 insufflation	118.76	5.95	128.78	5.28	-14.34	0.006
30 min after CO2 insufflation	116.96	6.22	126.67	4.01	-14.28	0.005
60 min after CO2 insufflation	115.11	5.67	125.40	3.84	-15.96	0.002
90 min after CO2 insufflation	113.51	5.56	123.58	3.73	-16.10	<0.001
After release of CO2	105.67	5.26	120.04	4.72	-16.48	<0.001

Baseline DBP comparison between Group I and Group II was not significant (p > 0.05). In Group I, DBP remained within the baseline throughout the study period, whereas in Group II, there was a rise in DBP during tracheal intubation and PNO insufflation, and it remained increased throughout the study period. The readings were found to be significantly lower in Group I (clonidine) than in Group II (normal saline). The p-value was <0.05 in most of the readings, which was statistically significant. Comparison of DBP is shown in Table 5.

**Table 5:** Diastolic blood pressure (DBP) recorded in both groups at specified timings Data are presented as mean ± SD. Independent samples t-test showed significantly lower DBP in Group I compared to Group II at multiple intraoperative time points (p < 0.05), while baseline values were comparable.

Diastolic Blood Pressure	Group - I		Group - II		Test Statistic t	P-value
	Mean	± SD	Mean	± SD		
Baseline	70.36	3.83	70.38	3.56	-0.02	0.977
15 min of IV drug	65.44	3.70	70.33	3.20	-6.71	0.016
Post-intubation	66.62	3.79	72.67	3.33	-8.04	0.019
Prior to CO2 insufflation	66.31	3.91	73.69	3.34	-9.62	0.035
5 min after CO2 insufflation	70.09	3.70	75.24	3.93	-6.40	0.015
15 min after CO2 insufflation	70.20	3.73	75.44	4.21	-6.25	0.011
30 min after CO2 insufflation	69.69	3.58	74.27	4.05	-5.68	0.008
60 min after CO2 insufflation	68.62	3.40	72.78	3.85	-5.43	0.004
90 min after CO2 insufflation	67.80	3.05	71.62	3.79	-5.27	<0.001
After release of CO2	66.67	2.43	69.91	4.13	-4.53	<0.001

Baseline MAP comparison between Group I and Group II was not significant (p > 0.05). In Group I, MAP remained within the baseline throughout the study period, whereas in Group II, there was a rise in MAP during tracheal intubation and PNO insufflation, and it remained increased throughout the study period. The readings were found to be significantly lower in Group I (clonidine) than in Group II (normal saline). P-value was <0.05, which was statistically significant in most of the readings. Comparison of MAP in both groups is shown in Table 6.

**Table 6:** Mean arterial pressure (MAP) recorded in both groups at specified timings Data are presented as mean ± SD. P < 0.05 is considered statistically significant. Independent samples t-test showed significantly lower MAP in Group I compared to Group II at multiple intraoperative time points (p < 0.05), while baseline values were comparable.

Mean Arterial Pressure	Group - I		Group - II		Test Statistic t	P-value
	Mean	± SD	Mean	± SD		
Baseline	86.64	4.29	86.76	3.84	-0.14	0.897
15 min of IV drug	81.18	4.19	86.58	3.64	-6.53	0.034
Post-intubation	82.78	4.20	93.24	3.19	-13.28	0.028
Prior to CO2 insufflation	82.16	4.08	92.02	3.29	-12.63	0.014
5 min after CO2 insufflation	86.18	4.01	94.40	3.59	-10.23	0.012
15 min after CO2 insufflation	86.40	3.74	95.53	3.14	-12.54	0.007
30 min after CO2 insufflation	85.38	3.68	93.78	2.95	-11.95	0.004
60 min after CO2 insufflation	84.11	3.28	92.42	2.93	-12.67	0.002
90 min after CO2 insufflation	83.09	3.08	90.91	2.99	-12.21	<0.001
After release of CO2	79.62	2.27	87.73	3.55	-12.91	<0.001

The incidence of side effects between the two groups is shown in Table 7. Bradycardia was observed in three patients (6.7%) in Group I, whereas no cases were reported in Group II, but this difference was not statistically significant (p = 0.242). Hypertension was not observed in Group I but occurred in 12 patients (26.7%) in Group II, which was found to be highly statistically significant (P < 0.001). Similarly, tachycardia was not observed in Group I but was noted in eight patients (17.8%) in Group II, showing a statistically significant difference (p = 0.006). No episodes of hypotension or postoperative vomiting were observed in either group.

**Table 7:** Side effects Values are expressed as frequency (%). n = number of patients in each group; N = total number of patients. Fisher’s exact test was used. P < 0.05 = statistically significant; P < 0.001 = highly significant.

Side Effects	Group I (n=45)	Group II (n=45)	Total (N=90)	P-value
Bradycardia	3 (6.7%)	0 (0.0%)	3 (3.3%)	0.242
Hypertension	0 (0.0%)	12 (26.7%)	12 (13.3%)	<0.001
Tachycardia	0 (0.0%)	8 (17.8%)	8 (8.9%)	0.006

Table 8 illustrates the comparison of the Ramsay Sedation Score, assessed using the Ramsay Sedation Scale (RSS), among the study groups [13]. A higher sedation score was observed in Group I compared to Group II postoperatively, but it never approached the asleep score of the Ramsay sedation scale at any time. The p-value for the Ramsay sedation scale was 0.157, which was statistically insignificant. 

**Table 8:** Ramsay Sedation Score Group I: Patients receiving intravenous clonidine (2 mcg/kg); Group II: Patients receiving intravenous normal saline (placebo). Ramsay Score ≤ 2 indicates awake or light sedation; Ramsay Score ≥ 3 indicates adequate sedation. Chi-square test was used. P < 0.05 was considered statistically significant.

Group	Ramsay Score ≤ 2 n (% )	Ramsay Score ≥3 n (%)	Total (n)	P-value
I	35 (78%)	10 (22%)	45	0.157
II	40 (89%)	5 (11%)	45	

## Discussion

The prospective, randomized, double-blind controlled trial demonstrated that intravenous (IV) clonidine administered at a dose of 2 µg/kg, 15 minutes before induction of anesthesia, significantly attenuated the hemodynamic responses associated with laryngoscopy, tracheal intubation, and carbon dioxide (CO₂) pneumoperitoneum in patients undergoing laparoscopic hysterectomy. Patients receiving clonidine exhibited significantly lower heart rate (HR), systolic blood pressure (SBP), diastolic blood pressure (DBP), and mean arterial pressure (MAP) throughout the intraoperative period compared with those receiving placebo. Furthermore, the requirement for rescue antihypertensive therapy was lower in the clonidine group, indicating superior perioperative hemodynamic stability. The critical window of induction and initial insufflation typically represents the highest risk period for sympathetic spikes, and our findings suggest that a pre-induction bolus effectively blankets this response.

The beneficial effects observed in the present study are consistent with those reported by Gupta et al., who demonstrated that clonidine premedication provided superior perioperative hemodynamic stability during laparoscopic surgery when compared with pregabalin [1]. While pregabalin targets the voltage-gated calcium channels to modulate neurotransmitter release, clonidine's central action appears to offer more robust intraoperative control against acute hypertensive surges. Tripathi et al. also reported that intravenous clonidine effectively attenuated the pressor response to laryngoscopy, tracheal intubation, and pneumoperitoneum [2]. Notably, their parallel dose-finding randomized controlled trial demonstrated that while a lower dose (1 µg/kg ) blunted the stress response to pneumoperitoneum alone, only the (2 µg/kg ) IV dose successfully provided consistent, comprehensive stability across both the intubation and extubation phases, which strongly reinforces the clinical choice of our titrated dosing protocol. These collective findings validate the efficacy of intravenous clonidine as a predictable anesthetic adjunct during laparoscopic procedures.

Carbon dioxide pneumoperitoneum produces significant cardiovascular alterations by increasing intra-abdominal pressure and stimulating sympathetic nervous system activity, resulting in increased systemic vascular resistance and arterial pressure [3,4]. These effects are further augmented by the steep Trendelenburg position commonly used during gynecological laparoscopy, which increases venous return and myocardial wall stress [4]. Naude et al. demonstrated that pneumoperitoneum is associated with increased systemic vascular resistance, elevated arterial pressure, and alterations in cardiac output during laparoscopic surgery [3]. Leonard and Cunningham likewise emphasized that maintaining hemodynamic stability during laparoscopic procedures remains a major anesthetic challenge because of the combined effects of pneumoperitoneum and patient positioning [4]. The attenuation of these hemodynamic responses observed in the present study is therefore consistent with the centrally mediated sympatholytic action of clonidine.

Clonidine is a selective α2-adrenergic receptor agonist that decreases central sympathetic outflow from the locus coeruleus, inhibits presynaptic norepinephrine release at peripheral nerve endings, and reflexively enhances vagal activity, thereby reducing heart rate and arterial blood pressure. Gregoretti et al. highlighted the perioperative advantages of clonidine, including improved cardiovascular stability, reduced volatile and intravenous anesthetic requirements, and a significant attenuation of the surgical stress response [5]. In the present study, these specific pharmacological mechanisms were clinically reflected by the consistently lower, more stable heart rate and blood pressure values observed throughout the duration of pneumoperitoneum, preventing the rapid oscillations that often complicate laparoscopic management.

The present findings are also highly comparable with those reported by Kalra et al., who demonstrated significantly lower heart rate and arterial pressure following intravenous clonidine administration during laparoscopic cholecystectomy [6]. Ray et al. similarly observed improved intraoperative hemodynamic stability together with reduced volatile anesthetic consumption following clonidine premedication, highlighting its role as a minimum alveolar concentration (MAC)-sparing agent [7]. Joris et al. further demonstrated that clonidine attenuated both the hemodynamic and endocrine responses induced by laparoscopic surgery, confirming that its benefits extend beyond macro-hemodynamics to cellular stress preservation [8]. Likewise, Laisalmi et al. reported enhanced cardiovascular stability with reduced anesthetic and opioid requirements in patients receiving clonidine, which has favorable implications for reducing opioid-related side effects postoperatively [9]. Singh and Arora also observed reduced perioperative hemodynamic fluctuations and improved postoperative analgesia following oral clonidine premedication [10].

Aho et al. demonstrated that clonidine effectively attenuated both hemodynamic and neuroendocrine responses during laparoscopic hysterectomy [11]. Similarly, Roy et al. reported improved perioperative cardiovascular stability in patients undergoing laparoscopic hysterectomy after intravenous clonidine administration [12].

In addition to its macro-cardiovascular effects, clonidine modulates the neuroendocrine axis. Yotsui demonstrated that clonidine reduced sympathetic hyperactivity during laparoscopic surgery, although activation of the hypothalamic-pituitary-adrenal (HPA) axis was not completely abolished, indicating that while catecholamine surges are blunted, upstream pathways may follow a distinct trajectory [14]. However, Sahajananda and Rao demonstrated significantly lower perioperative plasma cortisol concentrations following intravenous clonidine administration during laparoscopic surgery [15]. This biological mechanism is supported by matching biochemical RCTs in laparoscopic cohorts, which demonstrated that a pre-induction IV dose of clonidine limited the peak surge of plasma cortisol to a level two-to-three times lower than that observed in placebo controls at the end of surgery. Although plasma cortisol and catecholamine concentrations were not directly measured in the present study, the highly stable, improved macro-hemodynamic profile observed is entirely consistent with this established hormone-damping literature.

The incidence of adverse effects in the present study was remarkably low, underscoring the therapeutic safety margin of a 2 µg/kg dose. Bradycardia occurred in three patients receiving clonidine and responded promptly and completely to a single dose of atropine, while no patient developed clinically significant, prolonged hypotension or excessive postoperative sedation in the post-anesthesia care unit (PACU). Conversely, severe intraoperative hypertension and tachycardia requiring rapid-acting pharmacological intervention were significantly more frequent in the placebo group, exposing those patients to unnecessary cardiovascular stress. Similar safety profiles have been reported by Singh et al. [16], Bhandari et al. [17], Desai et al. [18], and Mali et al. [19], who collectively concluded that clonidine is generally well tolerated, with mild, manageable bradycardia representing the most common, easily treatable adverse effect.

The findings of the present randomized controlled trial have important clinical implications for modern anesthetic practice. Attenuation of perioperative hemodynamic responses may reduce myocardial oxygen demand and overall cardiovascular stress during laparoscopic surgery, a factor that is particularly vital in optimizing outcomes for patients with subclinical cardiovascular vulnerabilities. Furthermore, intravenous clonidine is highly inexpensive, widely available globally, straightforward to prepare and administer, and associated with a very favorable safety profile. Therefore, it may be considered an exceptionally effective and accessible premedicant for improving perioperative hemodynamic stability in patients undergoing laparoscopic hysterectomy.

Limitations of the study

This trial has several limitations. First, it was conducted at a single tertiary care center with a modest sample size, which may limit the broader generalizability of the findings across diverse clinical settings. Second, the protocol only included patients with American Society of Anesthesiologists (ASA) physical status I and II, meaning the results may not apply to elderly populations or patients with significant pre-existing cardiovascular disease. Third, the study focused entirely on intraoperative hemodynamics and did not evaluate secondary recovery metrics, such as postoperative analgesic requirements or patient satisfaction. Finally, biochemical markers of the surgical stress response, such as plasma cortisol or catecholamine concentrations, were not measured to objectively correlate with the clinical findings.

Future multicenter randomized controlled trials with larger sample sizes and higher-risk patient populations are warranted to validate these findings and further establish the role of intravenous clonidine in laparoscopic surgery.

## Conclusions

Intravenous clonidine at a dose of 2 µg/kg, administered 15 minutes before induction, was effective in reducing the hemodynamic responses associated with pneumoperitoneum during laparoscopic hysterectomy. Patients in the clonidine group showed better control of heart rate, systolic, diastolic, and mean arterial pressures compared to those receiving placebo. Furthermore, no significant postoperative sedation or major adverse effects were observed. These findings suggest that intravenous clonidine is a safe and useful premedication for achieving stable perioperative hemodynamics in patients undergoing laparoscopic hysterectomy under general anesthesia.
